# Economical Synthesis of ^13^C-Labeled Opiates, Cocaine Derivatives and Selected Urinary Metabolites by Derivatization of the Natural Products

**DOI:** 10.3390/molecules20045329

**Published:** 2015-03-25

**Authors:** Morten Karlsen, Huiling Liu, Jon Eigill Johansen, Bård Helge Hoff

**Affiliations:** 1 Department of Chemistry, Norwegian University of Science and Technology, Høgskoleringen 5, Trondheim NO-7491, Norway; E-Mail: Morten.Karlsen@chiron.no; 2 Chiron AS, Stiklestadveien 1, Trondheim 7041, Norway; E-Mails: Huiling.Liu@chiron.no (H.L.); Jon.Johansen@chiron.no (J.E.J.)

**Keywords:** ^13^C-labeled standards, heroin, codeine, morphine, 6-acetylmorphine, cocaine, benzoylecgonine, norcocaine, cocaethylene

## Abstract

The illegal use of opiates and cocaine is a challenge world-wide, but some derivatives are also valuable pharmaceuticals. Reference samples of the active ingredients and their metabolites are needed both for controlling administration in the clinic and to detect drugs of abuse. Especially, ^13^C-labeled compounds are useful for identification and quantification purposes by mass spectroscopic techniques, potentially increasing accuracy by minimizing ion alteration/suppression effects. Thus, the synthesis of [acetyl-^13^C_4_]heroin, [acetyl-^13^C_4_-methyl-^13^C]heroin, [acetyl-^13^C_2_-methyl-^13^C]6-acetylmorphine, [*N*-methyl-^13^C-*O*-metyl-^13^C]codeine and phenyl-^13^C_6_-labeled derivatives of cocaine, benzoylecgonine, norcocaine and cocaethylene was undertaken to provide such reference materials. The synthetic work has focused on identifying ^13^C atom-efficient routes towards these derivatives. Therefore, the ^13^C-labeled opiates and cocaine derivatives were made from the corresponding natural products.

## 1. Introduction

Drug abuse has a huge impact on individuals, their families, but also on society as a whole, due to loss of productivity, premature mortality, crime and healthcare costs [1]. However, narcotic drugs and psychotropic substances also have a variety of medical uses. Most importantly, opioids, such as codeine and morphine are indispensable for relieving moderate to severe pain [2,3]. Morphine is also used in the treatment of dyspnea [4,5], while codeine has been used for chronic cough [6] and diarrhea [7]. Heroin is a potent synthetic opiate analgesic synthesized from morphine by acetylation [8], but is banned for its high addictive character and finds little use in medicine. It is the most abused opiate and has become a worldwide rapidly increasing health problem. Some countries have started substitution therapy of heroin addicts, where the patient receives daily heroin doses under strict supervision [9].

**Figure 1:**
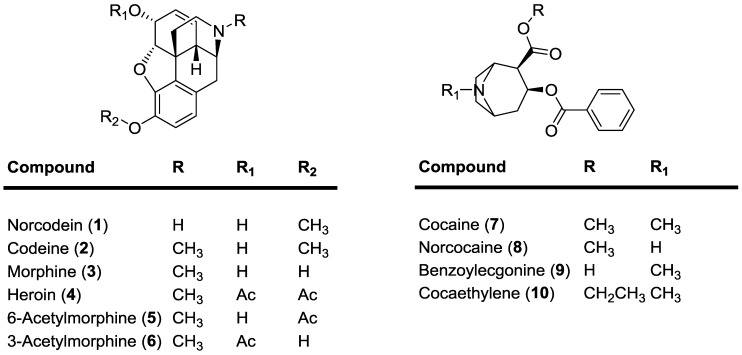
Opiate and cocaine derivatives focused on in this study.

*In vivo*, these drugs undergo metabolism to give a range of derivatives [10,11]. Thus, efficient analysis of both drugs and their metabolites in biological fluids are required in the fields of clinical toxicology and forensic toxicology, but also for workplace drug testing, testing of driving under the influence of drugs, doping analysis and rehabilitation programs. Moreover, in a therapy setting, there could be a need to track the use of such medicines and to confirm that the patients are using prescription medicines and not street material of varying quality. The most relevant opiate and cocaine derivatives are shown in Figure 1.

Drug analyses, which most preferably are performed with MS/MS techniques, require reference samples of the native drug, its main metabolites, alongside isotopically-labeled analogues. The labeled derivatives are used as a reference material to correct for error in the sample preparation and the effects of co-eluting substances that could alter or suppress the MS signal [12]. Deuterated derivatives can and have been used; however, a closer look at the published analytical methods for the quantification of these substances in bodily fluids [13,14] indicates that the protocols could benefit from using ^13^C-labeled internal standards (IS). In general, differences in extraction coefficients and retention time between the native drug and the deuterium analogue IS are likely to be seen when deuterium is situated in close proximity to heteroatoms [15,16]. Moreover, analyses of polar drugs and metabolites are often affected by coeluting matrix compounds.

In the case of cocaine (**5a**) and its metabolites, differences in retention times, extraction efficiency and matrix effects for deuterated and unlabeled material have been noted [13,17]. Even though these effects are relatively small (1%–20%), they could vary unpredictable from sample to sample, making quantification inaccurate. Especially, analysis of the cocaine metabolite benzoylecgonine (**5c**) with benzoylecgonine-d_8_ as IS is assumed challenging, since some of the deuterium labeling is on the ternary amino group, and the compound is relatively polar, eluting early in reverse phase systems. Furthermore, quantification of the zwitterionic polar morphine and its metabolites can be problematic using liquid chromatography [18,19]. Relatively large differences in matrix influence on the native and deuterium-labeled IS have sometimes been observed [13]. The matrix effects can be reduced by switching from ESI to atmospheric pressure chemical ionization (APCI) ionization, but this reduces the level of quantification significantly [14,18]. Thus, there is a need for alternative SIL IS to heighten the certainty in quantification of opiate and cocaine derivatives. This can be achieved using ^13^C-labeled analogues [17,18,19,20,21]. Analytical accuracy will increase by introducing more than one ^13^C-labeled atom, since overlap with the naturally abundant isotopes of the analyte being measured is minimized. Preferably, three ^13^C-labeled atoms should be incorporated. On this background, we herein disclosed the ^13^C-labeled synthesis of both the native compound and selected metabolites of opiates and the cocaine group of drugs to provide reference samples for quantification of drug content in biological samples.

## 2. Results and Discussion

Our aim was to synthesize ^13^C-labeled opiates and cocaine-derived standards useful in analytical chemistry. Initially, various total synthetic protocols were experimentally evaluated both in the case of morphine [20,21] and (−)-cocaine [22,23,24,25,26,27,28]. However, due to the complicated stereochemistry, long routes or the low yields experienced, an atom-efficient and economical synthesis could not be achieved. Thus, the ^13^C-labeled derivatives were made starting from the corresponding natural products.

### 2.1. Synthesis of ^13^C-Labeled Opiates

**Scheme 1:**
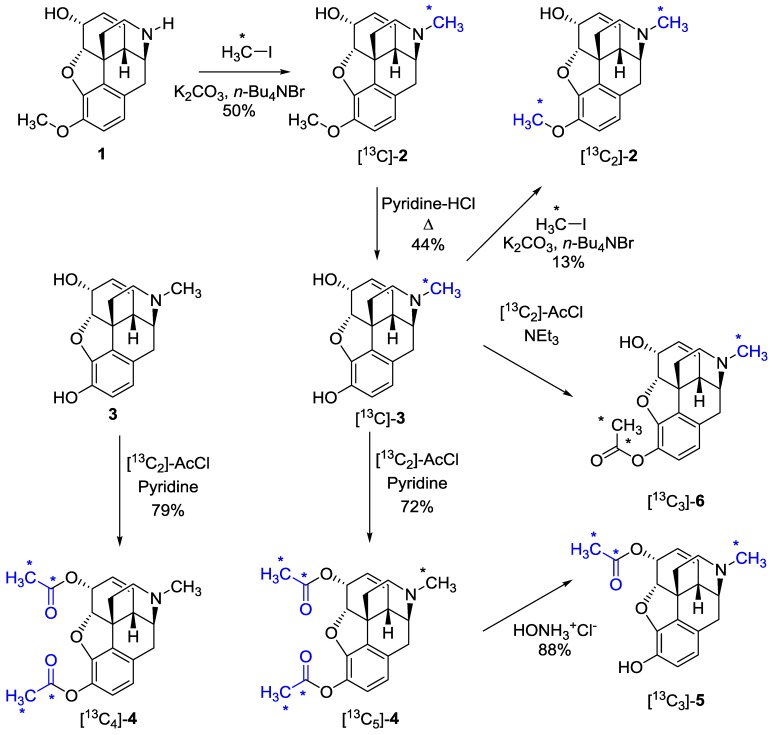
Synthesis of ^13^C-labeled codeine (**2**), morphine (**3**), heroin (**4**), 6-acetylmorphine (**5**) and 3-acetylmorphine (**6**).

Our preparation of the ^13^C-labeled opiates started with norcodeine (**1**), which can be synthesized from codeine in two steps [29,30]. Norcodeine (**1**) was alkylated by [^13^C]methyl iodide to form [methyl-^13^C]codeine ([^13^C]-**2**); see Scheme 1. Other methods for making normorphine or norcodeine tested were less successful or inconvenient, due to low yield, slow reaction [31] or hazardous reagents [32]. Compound [^13^C]-**2** was used to prepare [methyl-^13^C]morphine ([^13^C]-**3**) by *O*-demethylation using L-Selectride^®^ [30]. Morphine was isolated by extraction and then submitted to further purification by a sublimation process in a yield of 44%.

Our plan was to access [*N*-methyl-^13^C-*O*-methyl-^13^C]codeine ([^13^C_2_]-**2**), potentially useful as a standard, by alkylation of the morphine derivative, [^13^C]-**3**. This is challenging due to the three reactive groups of morphine (**3**), all capable of undergoing methylation, but several methods have been reported [33,34,35,36]. However, most strategies use reagents that are not commercially available, nor easily synthesized as their ^13^C-labeled analogues. Instead, a traditional alkylation with the reasonably-priced [^13^C]methyl iodide with tetrabutylammonium bromide as the phase transfer catalyst was employed. This allowed for short reaction times and yielded the double-labeled codeine derivative [^13^C_2_]-**2**, though in a low 13% yield.

[^13^C]-labeled heroin (**4**) can be produced as the [acetyl-^13^C_4_] analogue directly from morphine (**3**) or as [acetyl-^13^C_4_-methyl-^13^C]heroin from [^13^C]-**3**. The classical way to produce heroin is by acetylation of morphine (**3**) with excess acetic anhydride [8] or by the use of a catalyst to minimize the consumption of acetic anhydride [37]. We decided to use much lower priced [^13^C_2_]-acetyl chloride as the reagent for the esterification inspired by the dibenzoylation procedure reported previously [38]. The acylation proved successful using four equivalents of [^13^C_2_]acetyl chloride and gave [acetyl-^13^C_4_]heroin ([^13^C_4_]-**4**) and [acetyl-^13^C_4_-methyl-^13^C]heroin ([^13^C_5_]-**4**) in a 72% and 79% yield and high purity after crystallization from ethanol.

Upon administration, heroin rapidly hydrolyses to form 6-monoacetylmorphine (6-MAM, **5**), which is the main metabolite of heroin in man. Thus, 6-MAM is a good marker for heroin use [39]. [Acetyl-^13^C_2_-metyl-^13^C]-6-MAM (^13^C_3_-**5**) was synthesized by deacetylation of the heroin derivative, [^13^C_5_]-**4**, by reaction with hydroxylammonium chloride in ethanol [40]. This transformation gave a consistently high yield of [^13^C_3_]-**5** with only minor losses in the final recrystallization. The difference in reactivity of the 3-OH and 6-OH groups in morphine enables a selectively acetylation of morphine at the 3-position by using a weak base. The acetylation is fast, either with one equivalent of acetic anhydride or, as we found, with acetyl chloride. However, the unstable nature of 3-MAM (**6**) in solution and especially in protic solvents makes it nearly impossible to purify. It is reported that the benzoic salt is more stable [41], while mineral acid salts yield polymorphic crystals, which rapidly decompose. Thus, isolated [acetyl-^13^C_2_-metyl-^13^C]3-acetylmorphine ([^13^C_3_]-**6**) decomposed after short-term storage to yield a mixture of acetylated morphine derivatives. However, [^13^C_3_]-**6** could be clearly distinguished from [^13^C_3_]-**5** by its electron impact fragmentation profile.

### 2.2. [Phenyl-^13^C_6_]-Labeled Cocaine and Derivatives

**Scheme 2:**
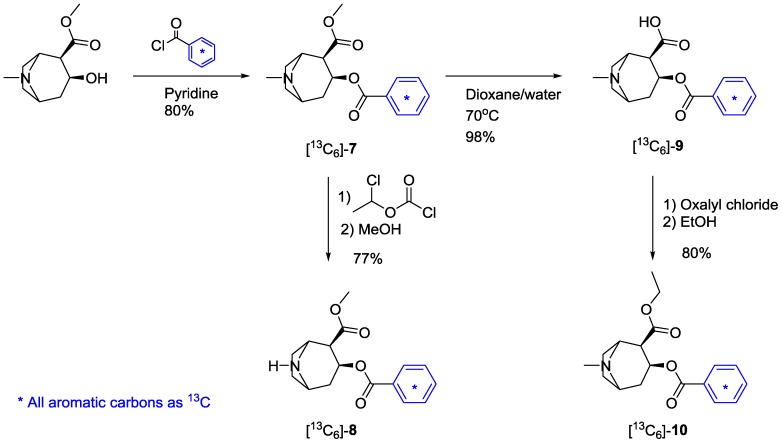
Synthesis of [phenyl-^13^C_6_]cocaine ([^13^C_6_]-**7**) and its ^13^C-labeled metabolites **8**–**10**.

Four cocaine derivatives were targeted, containing at least three incorporated ^13^C- atoms, namely (−)-cocaine (**7**), norcocaine (**8**), benzoylecgonine (**9**) and cocaethylene (**10**), starting from commercial (−)-cocaine (**7**). The chemistry utilized is shown in Scheme 2.

Hydrolysis of (−)-cocaine (**7**) gave ecgonine methyl ester hydrochloride, which was esterified with benzoyl-1,2,3,4,5,6-^13^C_6_ chloride in pyridine to yield [phenyl-^13^C_6_]cocaine ([^13^C_6_]-**7**). Labeled benzoyl chloride, in turn, was synthesized from benzaldehyde by a potassium permanganate oxidation in water/acetone followed by chlorination with thionyl chloride. To avoid hydrolysis during the evaporation of pyridine in the work-up, it was found crucial to quench the reaction with a saturated solution of sodium bicarbonate. [Phenyl-^13^C_6_]cocaine ([^13^C_6_]-**7**) was then used to synthesize [phenyl-^13^C_6_]norcocaine ([^13^C_6_]-**8**), [phenyl-^13^C_6_]benzoylecgonine ([^13^C_6_]-**9**) and [phenyl-^13^C_6_]cocaethylene ([^13^C_6_]-**10**). Demethylation of the tertiary amine in [^13^C_6_]-**7** was performed with 1-chloroethyl chloroformate in refluxing dichloroethane [42]. Removal of the reagent by evaporation and the addition of methanol with subsequent reflux yielded [phenyl-^13^C_6_]norcocaine ([^13^C_6_]-**8**) in a 77% yield after silica-gel chromatography [43]. To arrive at [phenyl-^13^C_6_]benzoylecgonine ([^13^C_6_]-**9**), the labeled cocaine derivative, [^13^C_6_]-**7**, as the free base, was gently hydrolysed at 70 °C in water/dioxane for two days. [44]. The product was isolated as its hydrate. Karl Fisher titration of the unlabeled material indicated this to be the tetrahydrate.

Cocaethylene (**10**) is an encountered metabolite when (−)-cocaine is co-administrated with ethanol [45]. [Phenyl-^13^C_6_]cocaethylene ([^13^C_6_]-**10**) was produced from [^13^C_6_]-**9** by chlorination with oxalyl chloride and esterification with ethanol in an 80% yield over two steps [46].

### 2.3. Analysis of the Products

The identity of the products was confirmed by co-elution with eutectic materials, GC/MS, high resolution mass spectroscopy and ^1^H- and ^13^C-NMR spectroscopy. Table 1 summarizes the ^1^H-NMR spectroscopic shifts of protons attached to the labeled carbons, their ^1^*J*_CH_ and ^2^*J*_CH_ coupling constants and the ^1^*J*_CC_ couplings, as observed from ^13^C-NMR spectroscopy.

**Table 1:** Selected NMR spectroscopic data for the ^13^C-labeled compounds, **2**–**6**.

Substance	Identity	^1^H-Shift (ppm)/Multiplicity	^1^*J*_C,H_ (Hz)	^2^*J*_C,H_ (Hz)	^1^*J*_C,C_ (Hz)
[*N*-Methyl-^13^C]codeine ([^13^C]-**2**)	N-CH_3_	2.46 (d)	133.1	-	-
[*N*-Methyl-^13^C-*O*-Methyl-^13^C]codeine ([^13^C_2_]-**2**)	N-CH_3_	2.46 (d)	133.1	-	-
	3-O-CH_3_	3.95 (d)	144.3	-	-
[Methyl-^13^C]morphine ([^13^C]-**3**)	N-CH_3_	2.46 (d)	133.1	-	-
[Acetyl-^13^C_4_]heroin ([^13^C_4_]-**4**)	6-OAc	2.14 (dd)	129.6	7.1	60.0
	3-OAc	2.28 (dd)	129.9	6.8	60.7
[Acetyl-^13^C_4_-methyl-^13^C]heroin ([^13^C_5_]-**4**)	6-OAc	2.14 (dd)	129.7	7.0	60.1
	3-OAc	2.28 (dd)	130.2	7.0	60.1
	N-CH_3_	2.50 (d)	133.6	-	-
[Acetyl-^13^C_2_-methyl-^13^C]6-MAM ([^13^C_3_]-**5**)	6-OAc	2.17 (dd)	130.1	6.6	59.3
	N-CH_3_	2.53 (d)	134.9	-	
[Acetyl-^13^C_2_-methyl-^13^C]3-MAM ([^13^C_3_]-**6**)	3-OAc	2.30 (dd)	129.9	6.9	60.1
	N-CH_3_	2.52 (d)	134.2	-	

The ^1^*J*_CH_ coupling constants varied from 129.7 to 144.3 Hz depending on the chemical environment, while the ^2^J_CH_ in the acetyl groups was observed to be in the range of 6.6 to 7.1 Hz. Furthermore, only minor variations in ^1^*J*_CC_ coupling constants were observed. The chemical purity of the materials for the opiates and the cocaine derivatives were determined using HPLC with MS detection. Furthermore, it was ensured that melting points had a narrow range. The crude products contain some water. Destructive water content measured by Karl Fisher was not performed on the materials reported herein, but on unlabeled material synthesized the same way. Typically, the materials were dried for 4–6 h at 60–70 °C at 1.8 × 10^−2^ mbar.

**Figure 2:**
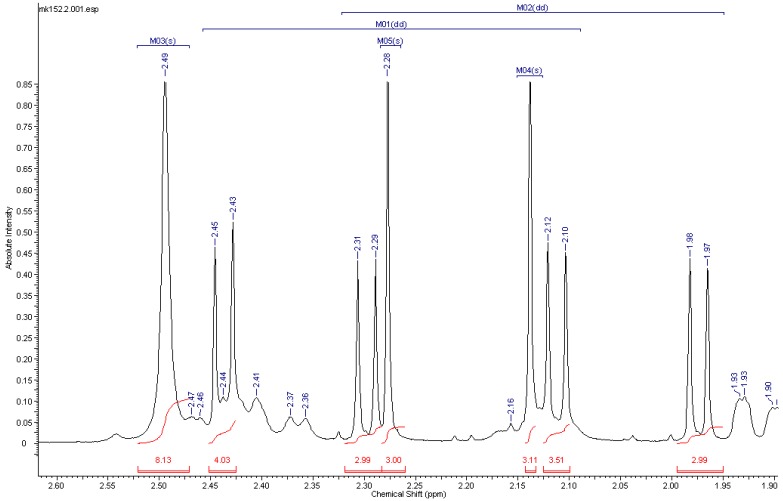
^1^H-NMR analysis of [acetyl-^13^C_4_]heroin ([^13^C_4_]-**4**) spiked with heroin (**4**).

The isomeric purity of the prepared materials solely relies on the isotopic purity of the starting material (>99% pure), as no exchange is possible in these reactions. In the case of the opiates, impurities from unlabeled material can in principle be detected by ^1^H-NMR by integration of acetyl signals. This is exemplified for a ^1^H-NMR analysis of [acetyl-^13^C_4_]heroin ([^13^C_4_]-**4**) spiked with heroin; see Figure 2. Whereas the unlabeled material gave two singlets for the acetyl protons, the signals from the labeled analogue give two doublets of doublets

## 3. Experimental Section

### 3.1. Chemicals and Analysis

Bulk solvents were purchased either from LabScan (Gliwice, Poland) or Merck (Darmstadt, Germany). Deuterated solvents were purchased from CDN Isotopes Inc. (Pointe-Claire, QC, Canada). All chemicals or reagents used were of the highest purity available and purchased from Sigma-Aldrich (Oslo, Norway) or Acros (Geel, Belgium). All solvents and chemicals were used as is without further purification unless otherwise stated. Anhydrous solvents were used as is and stored over activated molecular sieves. The silica-gel used for flash chromatography was Merck silica gel 60 (230–400 mesh). For chromatography, thin layer chromatography (TLC) silica gel 60F_254_ Merck plates/sheets were employed with visualization under UV light at 254 nm. ^1^H- and ^13^C-NMR spectra were recorded from Bruker Advance DPX instruments (400/100 MHz). Chemical shifts (δ) are reported in ppm relative to tetramethylsilane. Due to the high intensity of the ^13^C-labeled carbons as compared to those non-labeled, and multiple coupling, some NMR resonances were not detected. In this study, the non-labeled benzoic carbonyl signal at around 166 ppm was difficult to detect, experiencing extensive coupling by the neighboring ^13^C-isotopes. Isotopic purity and accurate mass determination in positive and negative mode on the final product was performed on a “Synapt G2-S” Q-TOF instrument from Waters with a resolution of 5 ppm. Samples were ionized by the use of an atmospheric pressure solids analysis probe (ASAP). No chromatography separation was used previous to the mass analysis. HPLC analysis was performed on an Agilent 1200 with atmospheric pressure chemical ionization (APCI)/electrospray ionization (ESI) multimode ionization. Acetonitrile as the mobile phase in combination with water buffered at pH 3 with formic acid. The column used was XBridge™ C18, 5 µm, 4.6 mm × 150 mm from Waters or Hilic Plus, 3.5 µm, 4.6 mm × 100 mm from Agilent. GC-MS analysis was performed on an Agilent 6890 with EI ionization, Agilent column HP5MS, 30 m, 0.25 mm internal diameter, 0.25-µm film. GC analysis of the final products’ free bases was performed as their trimethylsilyl derivatives (*N*,*O*-bis(trimethylsilyl)trifluoroacetamide derivatization). The identity of all products and intermediates were confirmed by co-elution with unlabeled materials and comparison of NMR spectra to confirm their structures. Water content was measured on unlabeled material using a headspace Karl Fischer Moisture Analysis 885 with a Metrohm 899 coulometer.

### 3.2. Synthesis of ^13^C-Labeled Opiates

#### 3.2.1. Synthesis of Norcodeine (**1**)

Codeine free base was azeotropically dried with refluxing toluene in a Dean-Stark trap for 24 h. Toluene was evaporated, and a codeine (10.0 g, 33.40 mmol) solution was made in chloroform (100 mL). To this solution, sodium bicarbonate (39.7 g, 473 mmol) was added, followed by the slow addition of methyl chloroformate (50.5 g, 534 mmol). After the exothermic part of the reaction had ceased, the reaction mixture was refluxed for 6 h. The reaction mixture was cooled to 22 °C and filtered, and the solvent and excess of reagent were removed under vacuum. Dry tetrahydrofuran (100 mL) was added to the residue, and L-Selectride (115 mL, 115 mmol, 1 M in tetrahydrofuran) was slowly added at 5 °C under argon. The reaction was the left to stir at 22 °C for 2 days. The reaction was quenched by the careful addition of water (50 mL) and hydrochloric acid (3 M, 100 mL). The reaction was then vigorously stirred at 0 °C in an ice/water bath. Solid norcodeine precipitated and was isolated by filtration. The precipitate was washed with ice-water (2 × 20 mL) and chilled acetone (50 mL). The crude product was then recrystallized from boiling water (110 mL), isolated by filtration and washed with acetone (3 × 30 mL). The yield of norcodeine after thoroughly drying was 8.46 g (29.65 mmol, 89%); purity: 98.9% (HPLC); mp 188.2–189.6 °C, literature (lit.) norcodeine [47] 186–187 °C; ^1^H-NMR (400 MHz, CHCl_3_) δ: 1.86–2.04 (m, 2 H), 2.65 (dt, *J* = 5.43, 2.84 Hz, 1 H), 2.67–2.85 (m, 2 H), 2.86–2.89 (m, 2 H), 2.90–3.04 (m, 2 H), 3.64–3.77 (m, 1 H), 3.86 (s, 3 H), 4.13–4.26 (m, 1 H), 4.89 (dd, *J* = 6.6, 1.0 Hz, 1 H), 5.27 (dt, *J* = 9.9, 2.7 Hz, 1 H), 5.69–5.81 (m, 1 H), 6.59 (d, *J* = 6.6, 1.0 Hz, 1 H), 6.69 (d, *J* = 8.1 Hz, 1 H); ^13^C-NMR (101 MHz, CHCl_3_) δ: 31.0, 36.2, 38.4, 40.9, 43.7, 52.0, 56.3, 66.2, 91.7, 113.0, 119.7, 126.9, 127.8, 130.9, 133.8, 142.3, 146.3.

#### 3.2.2. Synthesis of [*N*-Methyl-^13^C]codeine ([^13^C]-**2**)

Norcodeine (3.63 g, 12.7 mmol), [^13^C]methyl iodide (1.84 g, 12.9 mmol) and potassium carbonate (1.55 g, 38.2 mmol) in dry ethanol (50 mL) were heated at reflux for 3 hours, and the mixture was then cooled to room temperature. The solvent was evaporated under vacuum, and water was added (100 mL). The water phase was extracted with dichloromethane (3 × 50 mL), and the combined organic phases were washed with brine (40 mL) and dried over magnesium sulfate, filtered and evaporated to dryness. The crude product was first purified by crystallization from water (60 mL) and ethanol (6 mL) and then recrystallized from pentane (50 mL) and ethyl acetate (7 mL), yielding 1.89 g (6.29 mmol, 50%) of large orthorhombic crystals; mp 149.8–150.8 °C, lit. codeine [48] 150–152 °C; purity: of 97.3% (HPLC); GC-MS: 300.2 (100), 283.1 (11), 230.1 (23), 215.1 (15), 188.1 (11), 163.1 (24), 125.1 (10); ^1^H-NMR (400 MHz, CDCl_3_) δ: 1.89 (d, *J* = 12.4 Hz, 1H), 2.10 (td, *J* = 12.4, 4.8 Hz, 1H), 2.33 (dd, *J* = 18.8, 6.1, 2H), 2.43 (t, *J* = 12.4 Hz, 1H), 2.46 (d, *J* = 133.1 Hz, 3H), 2.71 (br.s., 1H), 2.92 (br.s., 1H), 3.06 (d, *J* = 18.7 Hz, 1H), 3.35–3.41 (m, 1H), 3.85 (s, 3 H), 4.16–4.22 (m, 1H), 4.90 (dd, *J* = 6.6, 1.0 Hz, 1 H), 5.30 (dt, *J* = 9.85, 2.65 Hz, 1H), 5.68–5.76 (m, 1H), 6.58 (d, *J* = 8.1 Hz, 1H), 6.67 (d, *J* = 8.1 Hz, 1H); ^13^C-NMR (101 MHz, CDCl_3_) δ: 20.4, 35.7, 40.6, 42.9, 43.0 (strong), 46.5, 56.3, 59.0, 66.3, 91.3, 112.9, 119.6, 128.1, 131.0, 133.5, 142.3, 146.3, 167.3; HRMS (ES^+^): calcd. for ^13^C_1_C_17_H_22_NO_3_ [M+H]^+^: 301.1633; found 301.1640.

#### 3.2.3. Synthesis of [*N*-Methyl-^13^C-O-methyl-^13^C]codeine ([^13^C_2_]-**2**)

[*N*-Methyl-^13^C]morphine ([^13^C]-**3**) (0.90 g, 3.34 mmol), potassium carbonate (0.29 g, 2.01 mmol) and tetrabutylammonium bromide (0.17 g, 5.26 mmol) were added to toluene (40 mL). The mixture was heated to 110 °C. A solution of [^13^C]methyl iodide (477 mg, 3.34 mmol) in toluene (10 mL) was then added drop-wise to the mixture. The reaction mixture was refluxed for 2 h, cooled to room temperature and quenched by the addition of water (20 mL). The two layers were separated, and the water layer was extracted with toluene (10 × 5 mL). The combined toluene layers were dried over magnesium sulfate. The mixture was filtered and evaporated yielding crude [^13^C_2_]-codeine (0.29 g), which was purified by preparative HPLC, yielding 126.3 mg (0.42 mmol, 13%) as white crystals; purity >99%; mp 149.3–150.7 °C, lit. codeine [48] 150–152 °C; GC-MS: 301.1 (100), 284.1 (11), 231.1 (23), 215.1 (15), 189.1 (11), 163.1 (25), 125.1 (10); ^1^H-NMR (400 MHz, CDCl_3_) δ: 1.89 (d, *J* = 12.4 Hz, 1H), 2.10 (td, *J* = 12.4, 4.8 Hz, 1H), 2.33 (dd, *J* = 18.7, 6.1, 2H), 2.43 (t, *J* = 12.4 Hz, 1H), 2.46 (d, *J* = 133.1, 3 Hz, 3H), 2.71 (br.s., 1H), 2.92 (br.s., 1H), 3.06 (d, *J* = 18.7 Hz, 1H), 3.66–3.69 (m, 1H), 3.95 (s, *J* = 144.3 Hz, 3H), 4.13–4.26 (m, 1H), 4.89 (dd, *J* = 6.6, 1.0 Hz, 1H), 5.27 (dt, *J* = 9.9, 2.7 Hz, 1H), 5.69–5.81 (m, 1H), 6.58 (d, *J* = 8.1 Hz, 1H), 6.67 (d, *J* = 8.1 Hz, 1H); ^13^C-NMR (101 MHz, CDCl_3_) δ: 31.0, 36.2, 38.4, 40.9, 43.0 43.7 (strong), 52.0, 56.3 (strong), 66.2, 91.7, 113.0, 119.7, 126.9, 127.8, 130.9, 133.8, 142.3, 146.3; HRMS (ES^+^): calcd. for ^13^C_2_C_16_H_22_NO_3_ [M+H]^+^: 302.1667; found 302.1669.

#### 3.2.4. Synthesis of [Methyl-^13^C]morphine ([^13^C]-**3**)

[*N*-Methyl-^13^C]codeine ([^13^C]-**2**) (1.70 g, 5.67 mmol) was treated with L-Selectride (2.5 equiv., 14.2 mL, 1.0 M in tetrahydrofuran) at reflux for 4 h. Water was carefully added (10 mL), and the tetrahydrofuran was evaporated under vacuum. Hydrochloric acid (2 M, 35 mL) was added, and the water phase was washed with chloroform (20 mL). The water phase was basified to pH 9 with ammonium hydroxide (28% solution), and the product was extracted with chloroform (3 × 50 mL). The combined organic extracts were washed with water pH adjusted to 9 with sodium bicarbonate (20 mL), dried over magnesium sulfate, filtered and evaporated to dryness. The crude product was purified by sublimation (110 °C, 2.5 × 10^−2^ mbar), yielding 708 mg (2.47 mmol, 44%); 98.5% (HPLC); mp 253.6–254.9 °C dec, lit. morphine [49] 255 °C; ^1^H-NMR (400 MHz, CDCl_3_) δ: 1.87 (d, *J* = 12.4 Hz, 1H), 2.09 (td, *J* = 12.4, 4.8 Hz, 1H), 2.34 (dd, *J* = 18.8, 6.1, 2H), 2.43 (t, *J* = 12.4 Hz, 1H), 2.46 (s, *J* = 133.1 Hz, 3 H), 2.69 (br.s., 1H), 2.92 (br.s., 1H), 3.06 (d, *J* = 18.7 Hz, 1H), 3.37–3.41 (m, 1H), 4.16–4.22 (m, 1H), 4.90 (dd, *J* = 6.6, 1.0 Hz, 1 H), 5.30 (dt, *J* = 9.85, 2.65 Hz, 1H), 5.68–5.76 (m, 1H), 6.58 (d, *J* = 8.1 Hz, 1H), 6.67 (d, *J* = 8.1 Hz, 1H), phenolic proton was not observed; ^13^C-NMR (101 MHz, CDCl_3_) δ: 31.0, 36.5, 38.6, 41.0, 42.8, 43.1 (strong singlet), 46.1, 56.3, 66.2, 91.7, 116.4, 120.0, 126.5, 127.7, 131.3, 145.5, 146.1; HRMS (ES^+^): calcd. for ^13^C_1_C_16_H_20_NO_3_ [M+H]^+^: 287.1477; found 287.1483.

#### 3.2.5. Synthesis of [Acetyl-^13^C_4_]heroin ([^13^C_4_]-**4**)

Dry morphine (**3**) as the free base (150 mg, 0.39 mmol) was dissolved in dry pyridine (10 mL) and then flushed with argon. [^13^C_2_]Acetyl chloride (62.8 mg, 0.78 mmol) was added slowly. The reaction temperature was then raised to 80 °C, and after one h, a new portion of [^13^C_2_]acetyl chloride (62.8 mg, 0.78 mmol) was added and the reaction stirred at 80 °C overnight. The mixture was cooled on an ice bath and quenched by the addition of sodium bicarbonate (50 mL). The organic phase was separated, and the water phase was extracted with dichloromethane (2 × 25 mL). The organic phases were combined, washed with water (10 mL) and dried over magnesium sulfate. After concentration, toluene (20 mL) was added to aid the evaporation of pyridine. The residue was washed with diethyl ether (10 mL) and recrystallized from ethanol (2 mL), which yielded [acetyl-^13^C_4_]heroin as needle crystals, which were washed with diethyl ether (10 mL) and dried. This gave 115 mg of free base (0.31 mmol, 79%); mp 170.7–171.0 °C, lit. heroin [50] 173 °C; purity of 99.5% (HPLC); ^1^H-NMR (400 MHz, CDCl_3_) δ: 1.91 (d, *J* = 10.6 Hz, 1H), 2.14 (dd, *J* = 129.6, 7.1 Hz, 3H), 2.28 (dd, *J* = 129.9, 6.8 Hz, 3H), 2.32–2.44 (m, 2H), 2.48 (s, 3H), 2.66 (br.s, 2H), 2.84 (br. S., 1H), 3.07 (d, *J* = 19.0 Hz, 1H), 3.42 (br. S., 1H), 5.04–5.24 (m, 2H), 5.44 (dt, *J* = 10.1, 2.40 Hz, 1H), 5.64 (dt, *J* = 10.1, 2.5 Hz, 1H), 6.60 (d, *J* = 8.1 Hz, 1H), 6.78 (d, *J* = 8.1 Hz, 1H); ^13^C-NMR (101 MHz, CDCl_3_) δ: 20.5, 20.6 (d, *J* = 60.7 Hz), 20.7 (d, *J* = 60.0 Hz), 34.9, 40.3, 42.7, 42.9, 46.6, 59.1, 68.0, 88.6, 119.4, 122.0, 128.6 (d, *J =* 2.2 Hz), 129.0–129.2 (m), 131.2–131.3 (m, 2C), 131.8–131.9 (m), 149.4, 168.3 (d, *J* = 60.7 Hz), 170.4 (d, *J* = 60.0 Hz); HRMS (ES^+^): calcd. for ^13^C_4_C_17_H_24_NO_5_ [M+H]^+^: 374.1789; found 374.1790.

#### 3.2.6. Synthesis of [Acetyl-^13^C_4_-metyl-^13^C]heroin ([^13^C_5_]-**4**)

Dry free-based [methyl-^13^C]morphine ([^13^C]-**3**) (150 mg, 0.39 mmol) was dissolved in dry pyridine (10 mL) and then flushed with argon. [^13^C_2_]Acetyl chloride (62.8 mg, 0.78 mmol) was added slowly. The reaction temperature was then raised to 80 °C, and after one hour, a new portion of [^13^C_2_]acetyl chloride (62.8 mg, 0.78 mmol) was added and the reaction stirred at 80 °C overnight. The mixture was cooled on an ice bath and quenched by the addition of sodium bicarbonate (50 mL). The organic phase was separated, and the water phase was extracted with dichloromethane (2 × 25 mL). The organic phases were combined, washed with water (10 mL) and dried over magnesium sulfate. After the solvent was evaporated, toluene (20 mL) was added, and the evaporation removed residues of pyridine. The residue was washed with diethyl ether (10 mL) and recrystallized from ethanol (2 mL), which yielded [^13^C_5_]-diacetylmorphine as needle-like crystals, washed with diethyl ether (10 mL) and dried. This gave 104 mg (0.28 mmol, 72%) of free base [^13^C_5_]-diacetylmorphine; mp 173.4–174.0 °C, lit. heroin [50] 173 °C; purity: 99.5% (HPLC); GC-MS: 374.2 (80), 330.2 (100), 313.2 (52), 269.1 (55), 207.1 (26), 163.1 (11), 147.1 (12);^1^H-NMR (400 MHz, CDCl_3_) δ: 1.93 (d, *J* = 12.4 Hz, 1H), 2.14 (dd, *J* = 129.7, 7.0 Hz, 3H), 2.28 (dd, *J* = 130.2, 7.0 Hz, 3H), 2.32–2.44 (m, 2H), 2.50 (d, *J* = 133.6 Hz, 3H), 2.67 (br.s., 2H), 2.89 (br.s., 1H), 3.08 (d, *J* = 19.0 Hz, 1H), 3.45 (br. s., 1H), 5.09–5.22 (m, 2H), 5.44 (dt, *J* = 10.0, 2.3 Hz, 1H), 5.65 (d, *J* = 9.9 Hz, 1H), 6.61 (d, *J* = 8.9 Hz, 1H), 6.79 (d, *J* = 8.3 Hz, 1H); ^13^C-NMR (101 MHz, CDCl_3_) δ: 20.6 (d, *J* = 60.1 Hz), 20.7, 20.7 (d, *J* = 59.3 Hz), 34.9, 39.4, 42.8 (strong singlet, 2C), 46.7, 59.2, 67.9, 88.5, 119.4, 122.1, 128.8, 128.9, 131.0, 131.2, 131.9, 149.4, 168.4 (d, *J* = 60.1 Hz), 170.5 (d, *J* = 60.1 Hz); HRMS (ES^+^): calcd. for ^13^C_5_C_16_H_24_NO_5_ [M+H]^+^: 375.1822; found 375.1826.

#### 3.2.7. Synthesis of [Acetyl-^13^C_2_-metyl-^13^C]6-acetylmorphine ([^13^C_3_]-**5**)

Compound [^13^C_5_]-**4** (150 mg, 0.4 mmol) was dissolved in ethanol (20 mL), and hydroxylammonium chloride (300 mg, 4.32 mmol) was added. The reaction mixture was stirred at 22 °C for 20 h. After evaporation of the solvent, the residue was dissolved in saturated sodium bicarbonate solution (50 mL) and extracted with chloroform (3 × 20 mL). The combined organic extracts were dried over magnesium sulfate, filtered and taken to dryness. The crude product was dissolved in acetonitrile (2 mL) and was slowly diluted with diethyl ether by diffusion. The product then crystallized as white needles, yielding after drying 112 mg (0.35 mmol, 88%); mp 193.7–194.6 °C; purity: 99.7% (HPLC); GC-MS: 330.2 (100), 269.1 (75), 216.1 (27), 163.1 (11), 147.1 (15); ^1^H-NMR (400 MHz, CDCl_3_) δ: 1.89 (d, *J* = 13.1 Hz, 1H), 2.17 (dd, *J* = 130.1, 6.6 Hz, 3H), 2.19 (br.s, 1H), 2.36 (br.s., 1H), 2.30–2.59 (m, 2H), 2.53 (d, *J* = 134.9 Hz, 3H), 2.70 (br.s., 2H), 3.05 (d, *J* = 19.2 Hz, 1H), 3.49 (br. s., 1H), 5.06–5.24 (m, 2H), 5.43 (dt, *J* = 10.1, 2.3 Hz, 1H), 5.63 (d, *J* = 10.1 Hz, 1H), 6.53 (d, *J* = 8.3 Hz, 1H), 6.67 (d, *J* = 8.1 Hz, 1H); ^13^C-NMR (101 MHz, CDCl_3_) δ: 20.9 (d, *J* = 59.3 Hz), 20.9, 34.8, 39.9, 42.7 (strong), 44.59, 46.8, 57.1, 88.5, 117.6, 119.7, 125.9, 128.0, 129.8, 129.3, 130.9, 140.6, 145.2, 170.5 (d, *J* = 59.3 Hz, strong); HRMS (ES^+^): calcd. for ^13^C_3_C_16_H_22_NO_4_ [M+H]^+^: 331.1649; found 331.1653.

#### 3.2.8. Synthesis of [Acetyl-^13^C_2_-metyl-^13^C]3-acetylmorphine ([^13^C_3_]-**6**)

[Methyl-^13^C]morphine ([^13^C]-**3**) (0.50 g, 1.29 mmol) was suspended in dry dichloromethane (30 mL). Triethylamine (0.44 g, 4.38 mmol) was added in one portion, whereupon the morphine base dissolved. [^13^C_2_]Acetyl chloride (1.42 mg, 1.77 mmol) in dry dichloromethane (10 mL) was added slowly to the reaction mixture. The reaction was kept at ambient temperature for 30 min and then refluxed for another 30 min before cooling in an ice bath and quenching with a saturated solution of sodium bicarbonate (50 mL). The organic phase was separated and the water phase extracted with dichloromethane (2 × 25 mL). The combined organic phases were washed with water (50 mL) and dried over magnesium sulfate. Evaporation of the solvent yielded the product as a clear oil. Diethyl ether (20 mL) was added and by-products settled as a white solid identified as [acetyl-^13^C_4_-methyl-^13^C]heroin. Isolation gave 379 mg (1.14 mmol, 89%) of [^13^C_3_]-**6** as a clear oil. GC-MS: 330.2 (100), 286.1 (95), 269.1 (15), 216.1 (26), 163.1 (33), 125.1 (12); ^1^H-NMR (400 MHz, CDCl_3_) δ: 1.87 (d, *J* = 12.4 Hz, 1H), 2.30 (dd, *J* = 129.9, 6.9 Hz, 3H), 2.32–2.43 (m, 2H), 2.52 (d, *J* = 134.2 Hz, 3H), 2.67 (br.s., 2H), 2.89 (br.s., 1H), 3.08 (d, *J* = 19.0 Hz, 1H), 3.32 (br. s., 1H), 4.19 (br.s, 1H), 5.01–5.25 (m, 2H), 5.25 (dt, *J* = 10.0, 2.3 Hz, 1H), 5.71 (d, *J* = 9.9 Hz, 1H), 6.61 (d, *J* = 8.9 Hz, 1H), 6.79 (d, *J* = 8.3 Hz, 1H); ^13^C-NMR (101 MHz, CDCl_3_) δ: 20.6 (d, *J* = 60.1 Hz), 20.9, 34.8, 39.9, 42.7 (strong), 44.59, 46.8, 57.1, 88.5, 117.6, 119.7, 125.9, 128.0, 129.8, 129.3, 130.9, 140.6, 145.2, 168.4 (d, *J* = 60.1 Hz). The product was contaminated with *ca*. 10% [acetyl-^13^C_4_-methyl-^13^C]heroin.

### 3.3. Synthesis of [Phenyl-^13^C_6_]-Labeled Cocaine Derivatives

#### 3.3.1. Synthesis of [Phenyl-^13^C_6_]cocaine ([^13^C_6_]-**7**) Hydrochloride

Thionyl chloride (2 mL) was added to benzoyl-1,2,3,4,5,6-^13^C_6_ acid (311 mg, 2.4 mmol) under argon atm. The reaction was warmed to 65 °C for 2 h. The excess reagent was evaporated under vacuum. To the crude benzoyl-1,2,3,4,5,6-^13^C_6_ chloride was added pyridine (2 mL), and the reagent was added slowly to a stirred solution of (−)-methylecgonine hydrochloride (413 mg, 1.8 mmol) in pyridine (5 mL) at 0 °C. The resulting solution was stirred at ambient temperature for 24 h and then cooled to 10 °C. The cooled solution was added to saturated solution of sodium bicarbonate, and the pyridine was removed under vacuum; then, toluene was added (5 mL) and evaporated. Water (20 mL) and ammonium hydroxide was added until the pH of the combined aqueous solution was 9. After the pH adjustment, the combined aqueous solution was extracted twice with chloroform (2 × 20 mL). The water phase can be acidified and cooled to precipitate unused benzoyl-1,2,3,4,5,6-^13^C_6_ acid. The combined chloroform extracts were dried over sodium carbonate, filtered and evaporated to yield a yellow oil. The oil was dissolved in *tert*-butyl methyl ether (10 mL), stirred with silica-gel (0.1 g), filtered and dried in a vacuum to give the product [^13^C_6_]-**7** as a clear oil. This was dissolved in diethyl ether, and HCl × isopropanol (5.5 M) was added until pH 4. The solid salt was filtered and washed with diethyl ether and recrystallized from ethanol and diethyl ether. The second crop of crystals was isolated upon concentration of the mother liquor. The total yield of the hydrochloride of [^13^C_6_]-**7** was 362 mg (1.05 mmol, 59%) with a purity of 99% (HPLC); mp. 199.1–200.0 °C, lit. cocaine hydrochloride [51] >197 °C; GC-MS: 309.2 (34), 278.1 (13), 199.1 (12), 182.1 (100), 111.1 (25), 82.1 (68); ^1^H-NMR (400 MHz, CD_3_OD) δ: 2.17–2.34 (m, 2H), 2.37–2.63 (m, 4H), 2.93 (s, 3H), 3.60–3.66 (m, 1H), 3.69 (s, 3H), 4.03–4.13 (m, 1H), 4.27 (m, 1H), 5.62 (dt, *J* = 10.7, 7.3 Hz, 1H), 7.21–7.55 (m, 1H), 7.61–7.96 (m, 2H), 8.07–8.37 (m, 2H); ^13^C-NMR (101 MHz, CD_3_OD) δ: 23.9, 25.1, 34.1, 39.7, 47.5, 53.6, 64.7, 65.3, 65.6, 128.8–131.5 (m, 5C), 133.7–136.1 (m, 1C), 174.32. The benzoate carbonyl signal at *ca*. 166 ppm was not detected; HRMS (ES^+^): calcd. for ^13^C_6_C_11_H_22_NO_4_ [M+H]^+^: 310.1750; found 310.1752.

#### 3.3.2. Synthesis of [Phenyl-^13^C_6_]norcocaine ([^13^C_6_]-**8**)

[Phenyl-^13^C_6_]cocaine ([^13^C_6_]-**7**) free base (80 mg, 0.26 mmol) was dissolved in 1,2-dichloroethane (5 mL), and 1-chloroethyl chloroformate (36 mg, 0.26 mmol) was added. The reaction was refluxed (80 °C) for 7 h. The reaction seems to have halted, and another equivalent of 1-chloroethyl chloroformate (36 mg, 0.26 mmol) was added and the reaction refluxed overnight. The mixture was evaporated to dryness under vacuum, and methanol (5 mL) was added before the reaction was refluxed for 2 h and basified with saturated sodium bicarbonate solution. The whole mixture was evaporated and the residue purified by flash chromatography on silica-gel with a basic mobile phase (240 mL dichloromethane,9 mL methanol, and 1 mL ammonium hydroxide), collecting 3-mL fractions. The product eluted in Fractions 5–10 yielded 59 mg (0.20 mmol, 77%); mp 80.9–82.0 °C, lit. [*N*-methyl-C-14]norcocaine [52] 82 °C; purity: 99% (HPLC); (GC-MS, TFA derivative): 391.2 (10), 360.2 (6), 322.2 (9), 280.1 (10), 263.1 (66), 232.1 (11), 194.1 (37), 164.1 (40), 111.1 (100), 83.1 (32) ^1^H-NMR (400 MHz, CD_3_OD) δ: 2.15–2.30 (m, 4H) 2.36 (dd, *J* = 9.0, 3.2 Hz, 2H) 3.55 (dd, *J* =7.2, 2.4 Hz, 1H) 3.68 (s, 3 H) 4.18–4.26 (m, 1H) 4.30–4.40 (m, 1H) 5.59 (td, *J* = 9.0, 7.4 Hz, 1H) 7.19–7.56 (m, 2H) 7.58–7.95 (m, 3H) 8.04–8.35 (m, 1H); ^13^C-NMR (101 MHz, CD_3_OD) δ: 26.0, 26.8, 32.8, 46.4, 53.4, 56.0, 57.1, 65.9, 126.8–132.2 (m, 5C), 133.3–139.0 (m, 1C), 174.0. The benzoate carbonyl signal at *ca*. 166 ppm was not detected; HRMS (ES^+^): calcd. for ^13^C_6_C_10_H_20_NO_4_ [M+H]^+^: 296.1594; found 296.1595.

#### 3.3.3. Synthesis of [Phenyl-^13^C_6_]benzoylecgonine ([^13^C_6_]-**9**)

[Phenyl-^13^C_6_]cocaine ([^13^C_6_]-**7**) free base (87 mg, 0.28 mmol) was dissolved in water (3 mL) and dioxane (3 mL). The resulting mixture was stirred at 70 °C for two days. The water/dioxane mixture was removed under reduced pressure, and the residue was washed with diethyl ether (5 mL), yielding 81 mg (0.27 mmol, 98%) of product as a white solid; mp. 156.5–157.6 °C, lit. benzoylecgonine [53] 191–192 °C, purity: 99% (HPLC); ^1^H-NMR (400 MHz, CDCl_3_) δ: 1.90–2.10 (m, 2H), 2.14–2.50 (m, 4H), 2.60 (s, 3H), 3.06 (dd, *J* = 6.6, 2.8 Hz, 1H), 3.71 (m, 2H), 5.37 (dt, *J* =10.7, 6.6 Hz, 1H), 7.03–7.41 (m, 2H), 7.50–7.92 (m, 2H), 8.05–8.45 (m, 1H); ^13^C-NMR (101 MHz, CDCl_3_) δ: 24.0, 25.0, 34.0, 38.3, 48.6, 61.0, 61.0, 64.3, 125.0–135.8 (6C), 173.0. The benzoate carbonyl signal at *ca*. 166 ppm was not detected; HRMS (ES^+^): calcd. for ^13^C_6_C_10_H_20_NO_4_ [M+H]^+^: 296.1594; found 296.1596.

#### 3.3.4. Synthesis of [Phenyl-^13^C_6_]cocaethylene (**10**)

[Phenyl-^13^C_6_]benzoylecgonine ([^13^C_6_]-**9**) (60 mg, 0.20 mmol) was added to oxalyl chloride (1 mL) and stirred for 30 min. Excess reagent was evaporated under vacuum, and dry ethanol (2 mL) was added. The reaction was then stirred over night before ethanol was removed under vacuum. Purification was by silica-gel flash chromatography eluting with a basic mobile phase (244 mL dichloromethane, 5 mL methanol and 1 mL ammonium hydroxide) and collecting 3-mL fractions. The product eluted in Fractions 10-16 yielded 52 mg (0.16 mmol, 80%); purity: 99% (HPLC); GC-MS: 323.2 (45), 278.2 (27), 212.2 (15), 196.1 (100), 111.1 (25), 94.1 (26), 82.1 (70); ^1^H-NMR (400 MHz, CD_3_OD) δ: 0.95 (t, *J* = 7.3 Hz, 3H), 2.19–2.32 (m, 2H), 2.37–2.62 (m, 4H), 2.93 (s, 3H), 3.62 (dd, *J* = 7.20, 2.2 Hz, 1H), 4.04–4.21 (m, 3H), 4.26 (d, *J* = 6.8 Hz, 1H), 5.61 (dt, *J* = 11.2, 7.0 Hz, 1H), 7.24–7.57 (m, 1H), 7.62–7.94 (m, 3H), 8.09–8.31 (m, 1H); ^13^C-NMR (101 MHz, CD_3_OD) δ: 12.5, 22.4, 23.5, 32.6, 38.2, 45.9, 62.1, 63.1, 63.9, 64.1, 127.1–130.5 (m, 5C) 132.1–134.9 (m, 1C), 172.46. The benzoic carbonyl signal at *ca*. 166 ppm was not detected; HRMS (ES^+^): calcd. for ^13^C_6_C_12_H_24_NO_4_ [M+H]^+^: 324.1907; found 324.1913.

## 4. Conclusions

LC/MS-MS-based drug quantification requires high quality, stable standards. To provide a reference sample for trace drug analysis of opiates, cocaine and selected metabolites in biological samples, the synthesis of [acetyl-^13^C_4_]heroin, [acetyl-^13^C_4_-methyl-^13^C]heroin, [acetyl-^13^C_2_-methyl-^13^C]6-acetylmorphine, [*N*-methyl-^13^C-*O*-metyl-^13^C]codeine and phenyl-^13^C_6_-labeled derivatives of cocaine, benzoylecgonine, norcocaine and cocaethylene has been performed. The materials were prepared from the natural products by installing the ^13^C labels by *N*- and *O*-methylation and acylation reactions.

## Supplementary Materials

Supplementary materials can be accessed at: http://www.mdpi.com/1420-3049/20/04/5329/s1.
