# Supplementary material for: Economical Synthesis of 13C-Labeled Opiates, Cocaine Derivatives and Selected Urinary Metabolites by Derivatization of the Natural Products
Source: Molecules. 2015 Mar 25;20(4):5329–45. doi: 10.3390/molecules20045329 (PMC6272324; doi:10.3390/molecules20045329)
Supplement: Supplementary file 1 [file molecules-20-05329-s001.pdf]

Supplementary Materials

^{1}H-NMR Spectra of Labelled Opiates

^{1}H-NMR of [N-Methyl-^{13}C-O-methyl-^{13}C]codeine ([^{13}C$_2$]-2):

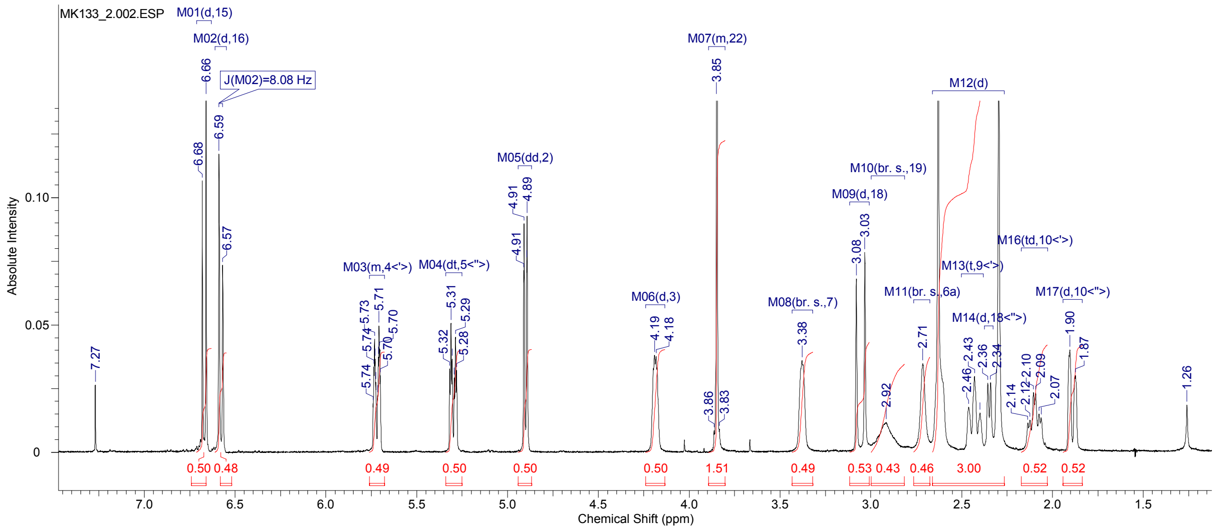

Figure S1. ^{1}H-NMR (400 MHz, $CDCl_3$) [*N*-methyl-^{13}C-O-methyl-^{13}C]codeine ([$^{13}C_2$]-**2**).

^{1}H-NMR of [Acetyl-^{13}C$_4$]heroin ([^{13}C$_4$]-4):

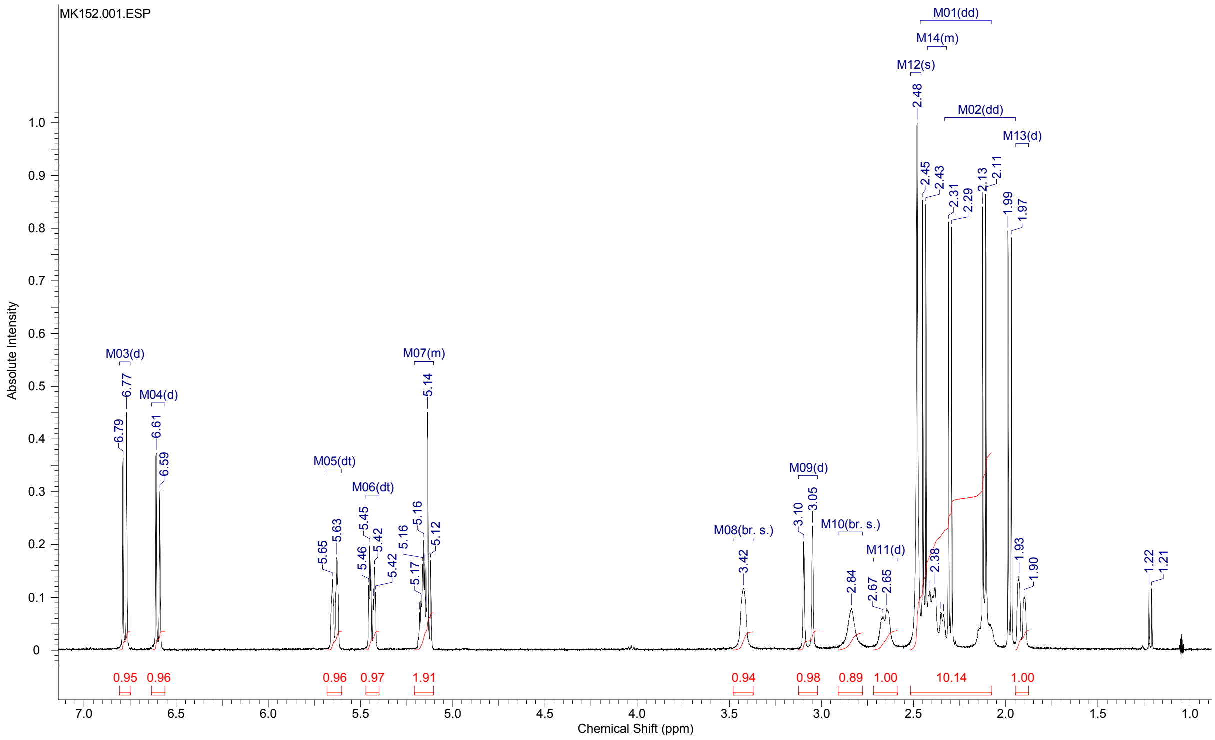

Figure S2. ^{1}H-NMR (400 MHz, $CDCl_3$) of [acetyl-$^{13}C_4$]heroin ([$^{13}C_4$]-**4**).

^{1}H-NMR of [Acetyl-$^{13}C_4$-metyl-^{13}C]heroin ([$^{13}C_5$]-4):

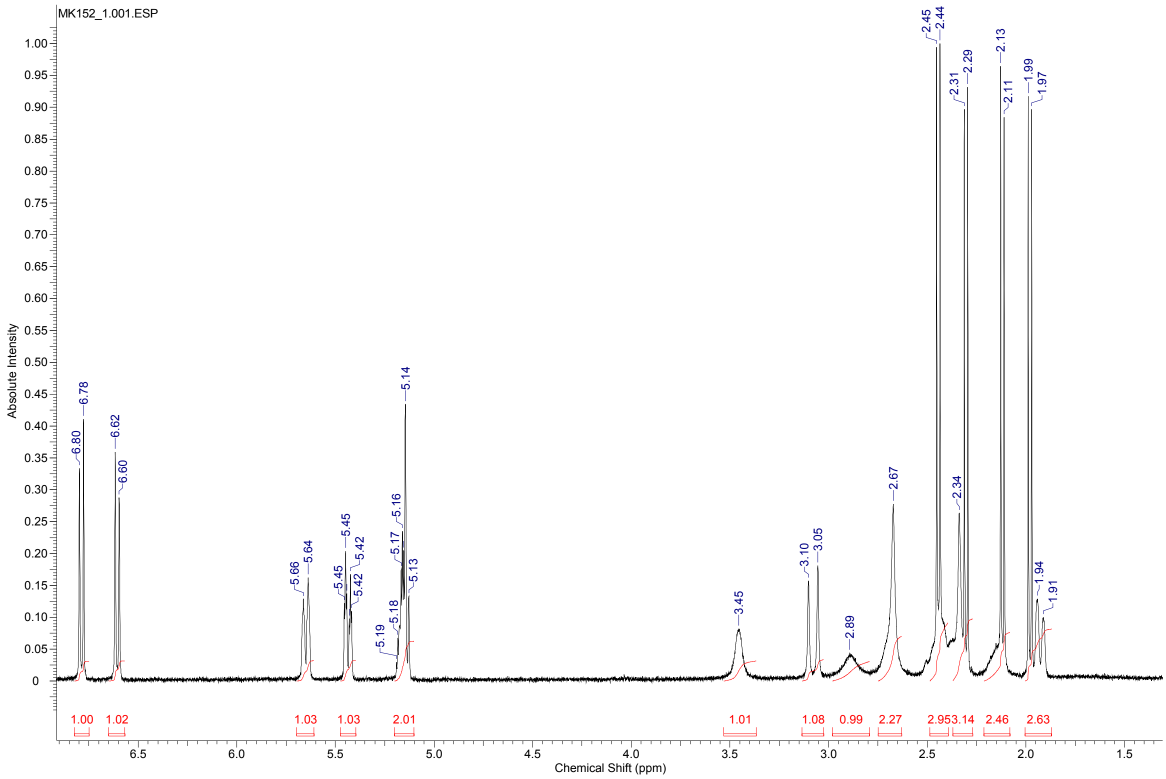

Figure S3. ^{1}H-NMR (400 MHz, $CDCl_3$) of [acetyl-$^{13}C_4$-metyl-^{13}C]heroin ([$^{13}C_5$]-**4**).

^{1}H-NMR of [Acetyl-$^{13}C_2$-metyl-^{13}C]6-acetylmorphine ([$^{13}C_3$]-5):

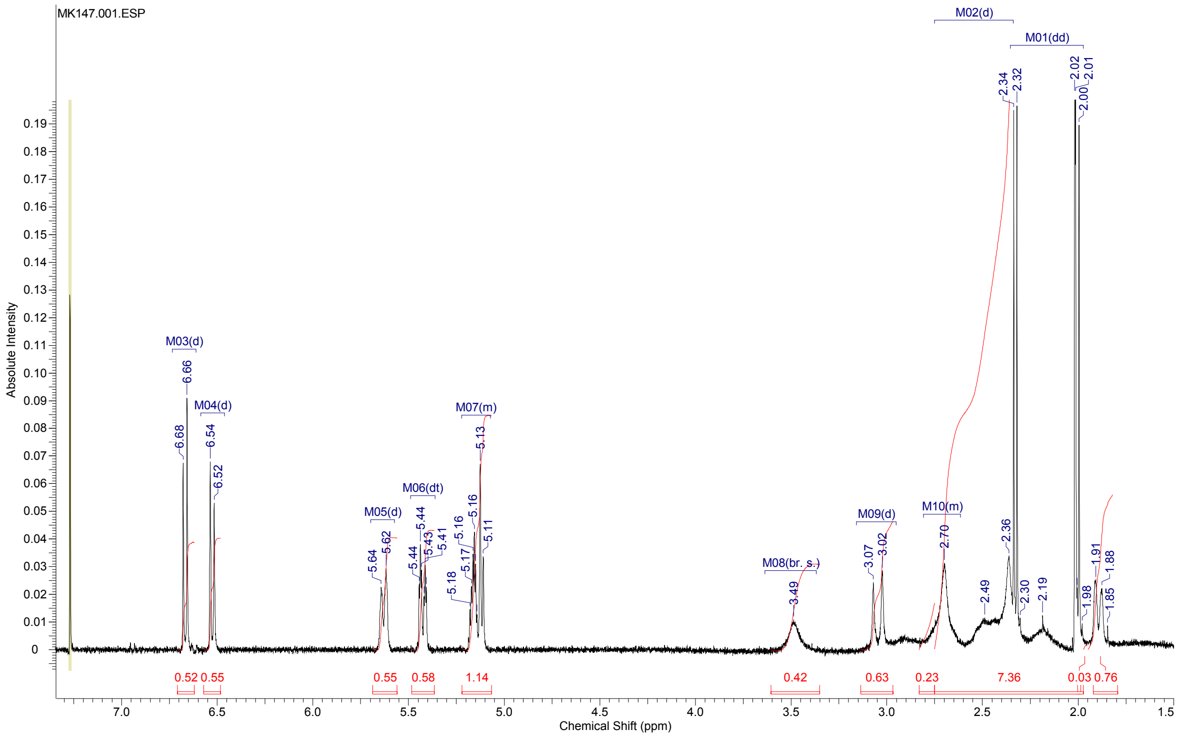

Figure S4. ^{1}H-NMR (400 MHz, $CDCl_3$) of [acetyl-$^{13}C_2$-metyl-^{13}C]6-acetylmorphine ([$^{13}C_3$]-**5**).

^{1}H-NMR of Labelled Cocaine Derivatives

^{1}H-NMR of [Phenyl-$^{13}C_6$]cocaine ([$^{13}C_6$]-7) hydrochloride:

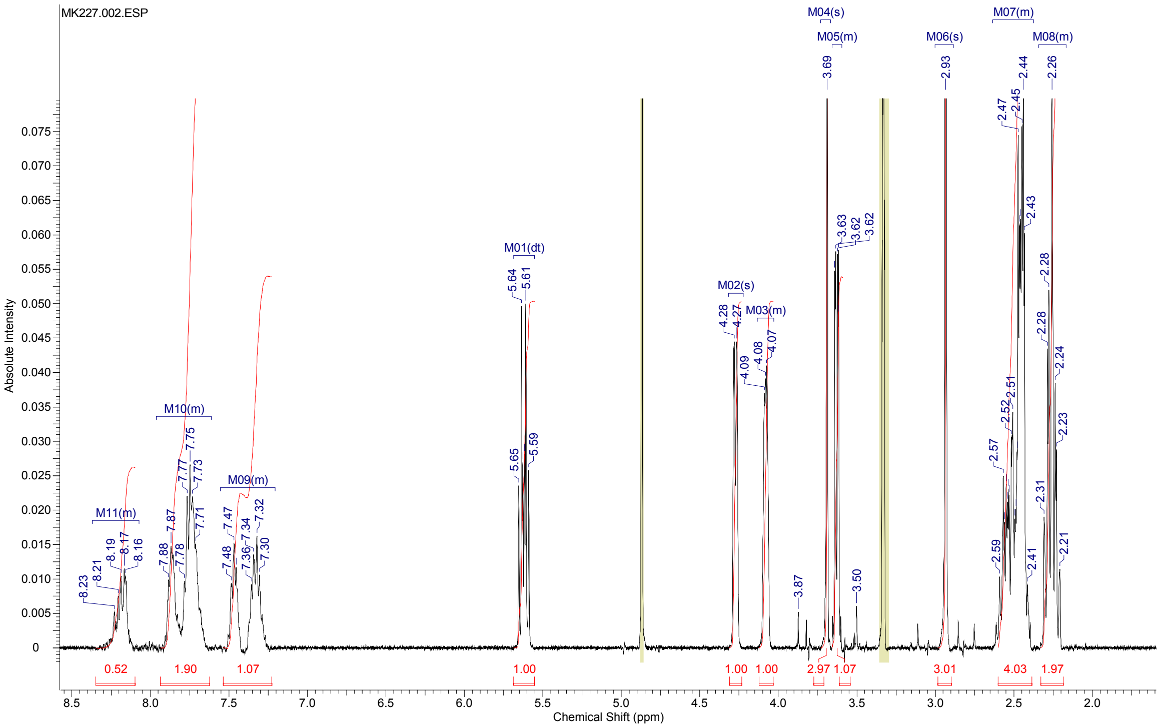

Figure S5. ^{1}H-NMR (400 MHz, CD_3OD) of [phenyl-$^{13}C_6$]cocaine ([$^{13}C_6$]-**7**) hydrochloride.

^{1}H-NMR of [Phenyl-$^{13}C_6$]norcocaine ([$^{13}C_6$]-8):

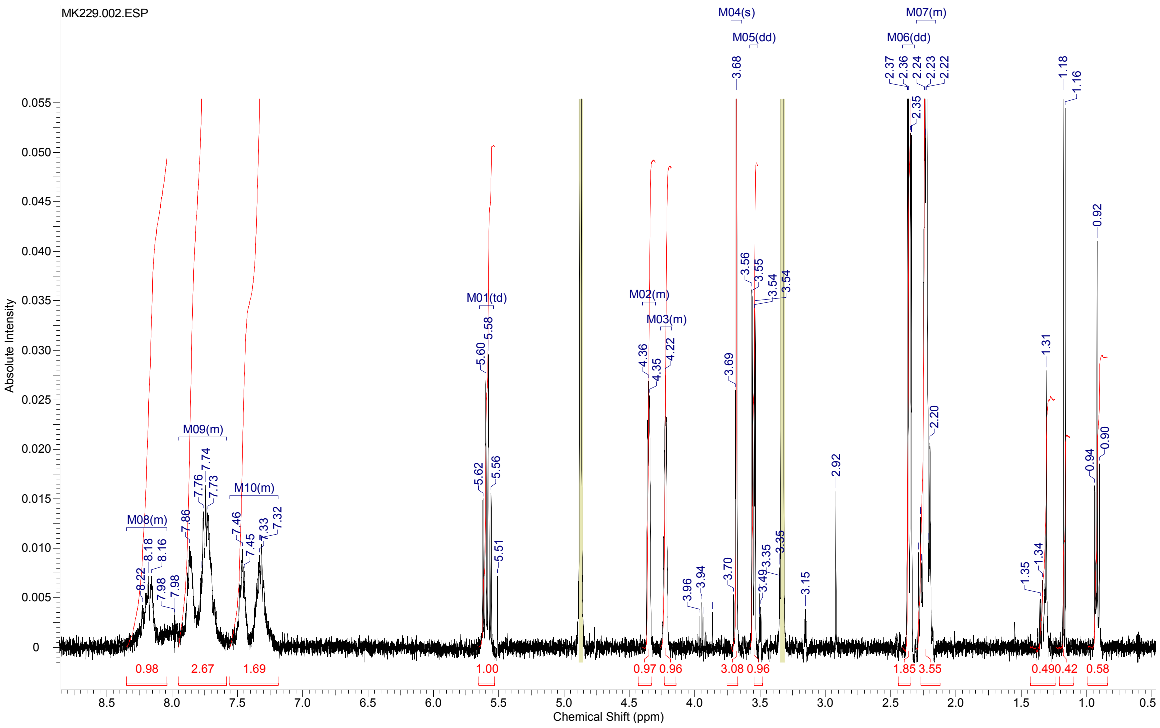

Figure S6. ^{1}H-NMR (400 MHz, CD_3OD) of [phenyl-$^{13}C_6$]norcocaine ([$^{13}C_6$]-**8**).

^{1}H-NMR of [Phenyl-^{13}C6]benzoylecgonine ([^{13}C6]-9)

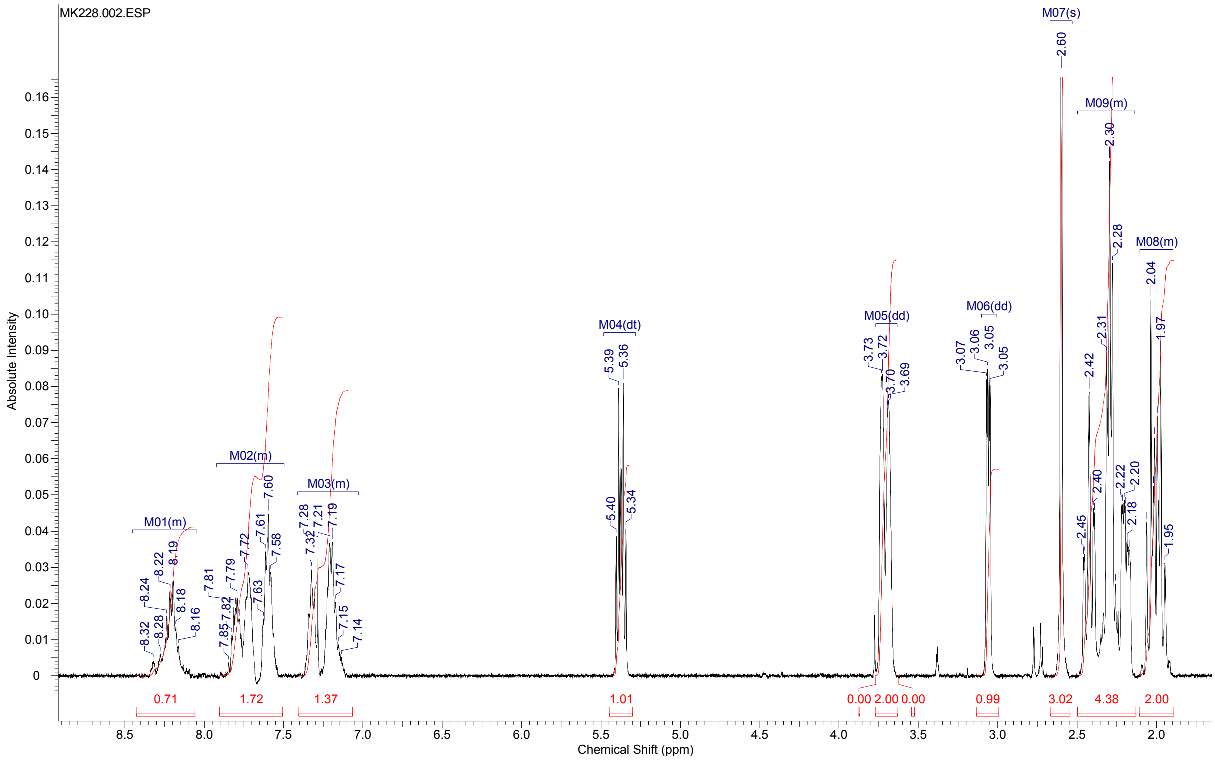

Figure S7. ^{1}H-NMR (400 MHz, $CDCl_3$) of [phenyl-$^{13}C_6$]benzoylecgonine ([$^{13}C_6$]-**9**).

^{1}H-NMR of[Phenyl-^{13}C6]cocaethylene (10):

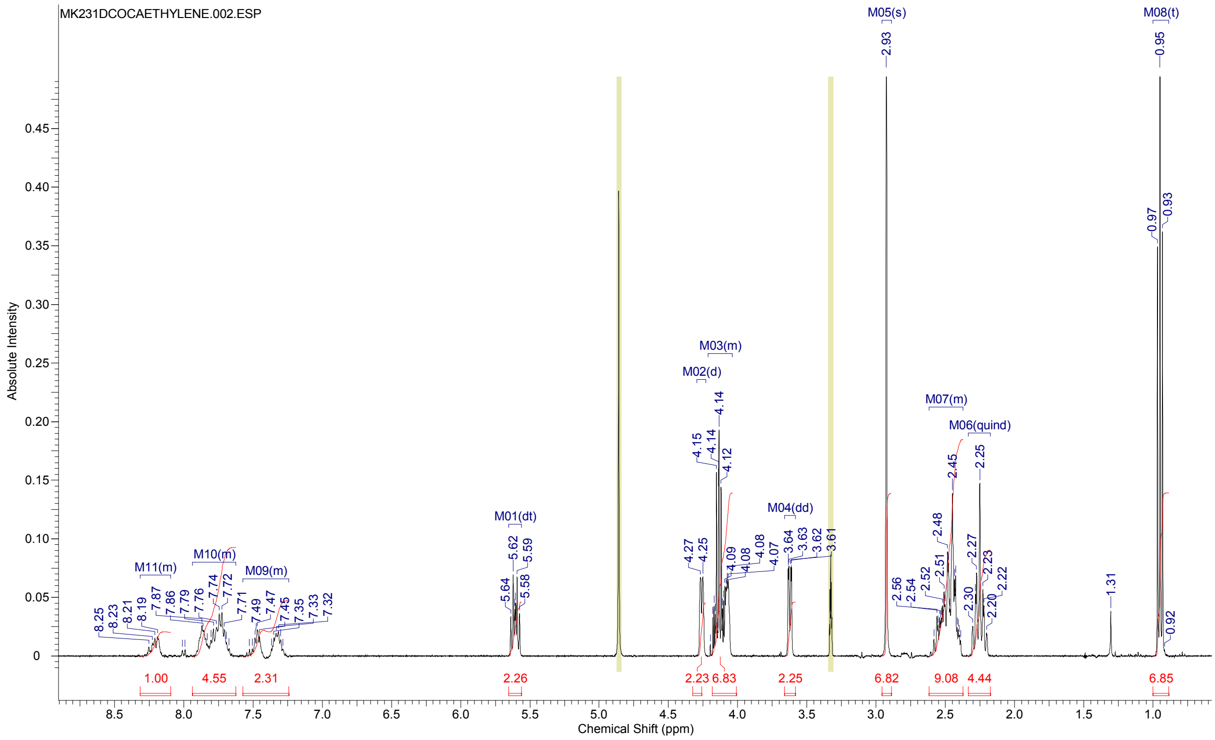

Figure S8. ^{1}H-NMR (400 MHz, CD_3OD) of [phenyl-$^{13}C_6$]cocaethylene (**10**).
